# microRNA‐1914, which is regulated by lncRNA DUXAP10, inhibits cell proliferation by targeting the GPR39‐mediated PI3K/AKT/mTOR pathway in HCC

**DOI:** 10.1111/jcmm.14705

**Published:** 2019-10-01

**Authors:** Liankang Sun, Liang Wang, Tianxiang Chen, Bowen Yao, Yufeng Wang, Qing Li, Wei Yang, Zhikui Liu

**Affiliations:** ^1^ Department of Hepatobiliary Surgery The First Affiliated Hospital of Xi'an Jiaotong University Xi'an China

**Keywords:** AKT, DUXAP10, GPR39, hepatocellular carcinoma, miR‐1914

## Abstract

Increasing studies have confirmed that abnormally expressed microRNAs (miRNAs) take part in the carcinogenesis as well as the aggravation of hepatocellular carcinoma (HCC). However, little information is currently available about miR‐1914 in HCC. Here, we first confirmed that miR‐1914 inhibition in HCC cell lines and tumour specimens correlates with tumour size and histological grade. In a series of functional experiments, miR‐1914 inhibited tumour proliferation and colony formation, resulting in cell cycle arrest and increased apoptosis. Moreover, miR‐1914 mediated its functional effects by directly targeting GPR39 in HCC cells, leading to PI3K/AKT/mTOR repression. Restoring GPR39 expression incompletely counteracted the physiological roles of miR‐1914 in HCC cells. In addition, down‐regulation of AKT phosphorylation inhibited the effects of miR‐1914 in HCC. Furthermore, the overexpression of lncRNA DUXAP10 negatively correlated with the expression of miR‐1914 in HCC; thus, lncRNA DUXAP10 regulated miR‐1914 expression and modulated the GPR39/PI3K/AKT‐mediated cellular behaviours. In summary, the present study demonstrated for the first time that lncRNA DUXAP10–regulated miR‐1914 plays a functional role in inhibiting HCC progression by targeting GPR39‐mediated PI3K/AKT/mTOR pathway, and this miRNA represents a novel therapeutic target for patients with HCC.

## 1 INTRODUCTION

Hepatocellular carcinoma (HCC) remains one of the most common malignancies and is the main cause of cancer‐related deaths worldwide.^1^ Despite the remarkable advancement in therapeutic strategies,^2^, ^3^ the prognosis remains unsatisfactory because only 10%‐37% of patients are suitable candidates for surgery because of late‐stage tumours or hepatic functional failure.^4^ Thus, further exploration of the mechanisms underlying HCC progression may help provide novel therapeutic targets.

As a set of evolutionarily conserved small non‐coding RNAs, microRNAs (miRNAs) have been identified as post‐transcriptional gene expression regulators that act via combining with complementary sequences within 3′‐untranslated regions (UTRs) of corresponding protein‐coding genes, eventually leading to mRNA degradation or translation inhibition.^5^, ^6^ Mounting studies have indicated that miRNAs are vital in diverse biological processes in HCC, including cell proliferation, cell cycle progression, apoptosis, differentiation and metastasis, because they can regulate tumour suppressors or oncogenes.^7^, ^8^ Therefore, specific miRNAs are known to be potential therapeutic, diagnostic and prognostic biological markers in HCC.^9^ miR‐1914, a newly identified cancer‐related microRNA, is aberrantly expressed and has become a predictor of cancer patients' survival.^10^, ^11^, ^12^ miR‐1914 facilitates cancer progression by repressing the expression of nuclear factor IX in colorectal cancer (CRC), and plasma miR‐1914 suppresses chemoresistance in CRC patients.^13^, ^14^, ^15^ Moreover, miR‐1914 is dysregulated in stomach cancer. However, the expression status and specific biological effects of miR‐1914, as well as the underlying molecular mechanism, require further exploration in HCC.

Herein, we revealed for the first time that the level of miR‐1914 was decreased in HCC and correlated with adverse prognostic features, confirming its critical role in HCC progression and potential as a target for treating HCC.

## 2 MATERIALS AND METHODS

### 2.1 Clinical samples and cell culture

In total, 134 HCC specimens and corresponding neighbouring non‐tumour samples were collected in our hospital from January 2006 to December 2009 with informed consent from all patients. These patients did not receive any chemotherapy, radiation therapy or immunotherapy before surgery. The Xi'an Jiaotong University Ethics Committee authorized the research based on the Declaration of Helsinki.

Normal immortalized human hepatocyte LO2 cells and HCC cell lines (Hep3B, HepG2, Huh7, MHCC‐97L and HCCLM3) (Chinese Academy of Sciences, Shanghai, China) were cultured in DMEM (Invitrogen, Carlsbad, USA) with 10 μL 10% FBS (Gibco, Grand Island, USA) at 37°C and 5% CO_2_.

### 2.2 RNA extraction and qRT‐PCR

qRT‐PCR was performed as reported previously.^16^, ^17^ Total RNA was extracted according to the protocol of TRIzol reagent (Invitrogen, Carlsbad, CA, USA). qPCR primers against miR‐1914 (HmiRQP0270), U6 (HmiRQP9001), GPR39 (HQP008237) and GAPDH (HQP006940) were obtained from GeneCopoeia (Guangzhou, China).

### 2.3 Immunohistochemical staining

Immunohistochemical staining (IHC) was conducted using an SABC kit (Maxim, Fuzhou, China) according to the manufacturer's instructions. In summary, HCC tissue sections were incubated with GPR39 (1:300, Abcam, USA) overnight at 4°C and then flushed with PBS and re‐incubated with biotinylated secondary antibodies (Goldenbridge, Zhongshan, China) according to the SP‐IHC assay instructions. The experiment was performed according to our previously reported protocol.^18^

### 2.4 Western blot analysis

Proteins were separated by SDS‐PAGE and transferred to PVDF membranes. The experiment was performed as previously described.^7^

### 2.5 RNA interference transfection

One negative control siRNA and GPR39 siRNA were designed and produced by GenePharm (Shanghai, China). MHCC‐97L and Hep3B cells (2 × 10^5^ per well) were transfected with 100 nmol/L siRNA.

### 2.6 Cell proliferation, cell cycle and apoptosis detection

The procedures for EdU, colony formation, cell cycle and apoptosis analyses were performed as reported previously.^7^

### 2.7 Luciferase reporter assay

The 3′‐UTR binding sequence of GPR39 and the associated mutated sequences were generated and inserted into the pmiR‐GLO dual‐luciferase miRNA target expression vector (Promega, Madison, WI, USA). Luciferase activity was determined according to previous protocols.^8^, ^19^

### 2.8 In vivo experiments

Female BALB/c nude mice (4‐6 weeks old and obtained from the Experimental Animal Center of Xi'an Jiaotong University School of Medicine, Xi'an) were utilized to establish a subcutaneously implanted tumour model. A lentiviral vector‐mediated miR‐1914 inhibitor (anti–miR‐inhibitor) and negative control (NC) and lentiviral vector‐mediated miR‐1914 and miR‐control were obtained from GeneCopoeia Inc (Guangzhou, China). Hep3B cells were transfected with anti–miR‐NC or anti–miR‐1914 vectors, and MHCC‐97L cells were transfected with miR‐1914 or miR‐control vectors. After transfection of the vectors, 5 × 10^6^ cells were mixed with 150 μL of Matrigel and injected subcutaneously into the flanks of nude mice. Tumour volumes were then monitored two‐dimensionally and calculated with the following formula: *V* (tumour volume: mm^3^) = 0.5 × [*w* (width: mm)]^2^ × *L* (long diameter: mm). Three weeks later, the mice were killed, and the weights of the xenograft tumour tissues were measured. These tumour tissues were then fixed for further histological analysis. All experiments were authorized by the Institutional Animal Care and Use Committee of Xi'an Jiaotong University.

### 2.9 Statistical analysis

To avoid systemic errors, each experiment was repeated more than three times. The results are displayed as the mean ± standard deviation. Student's *t* test or one‐way ANOVA (one‐way analysis of variance) followed by LSD post hoc test was conducted to compare the differences between two groups or more than two groups, respectively, with SPSS (SPSS 18.0; SPSS Inc, Chicago, IL, USA). A *P* value <.05 was regarded as statistically significant.

## 3 RESULTS

### 3.1 The expression level of miR‐1914 was decreased in HCC and correlated with adverse prognostic features

**Figure 1:**
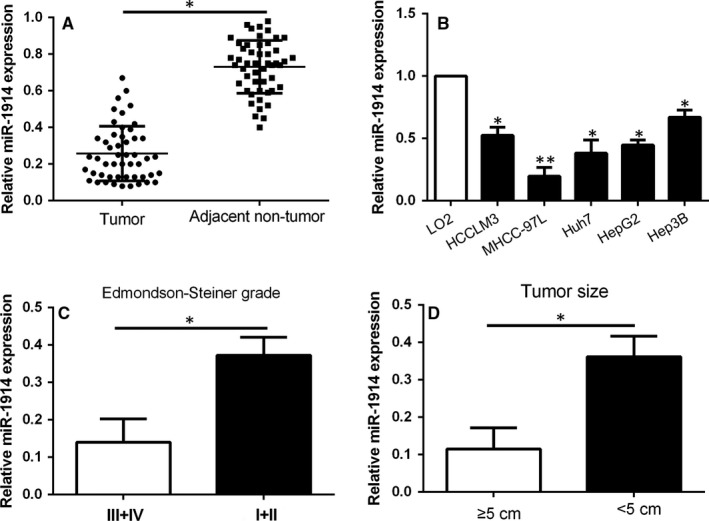
miR‐1914 is reduced in Hepatocellular carcinoma (HCC). Comparing the aberrant expression level of miR‐1914 among (A) HCC specimens versus tumour‐adjacent tissues, and (B) HCC cell lines versus LO2 cells; HCC tissues arising from different histological grade (C) and tumour size (D). *P < .05, **P < .01

To assess the expression level of miR‐1914 in HCC tissues, we selected 50 pairs of tissues randomly. The expression level of miR‐1914 was markedly lower in the HCC samples than in the corresponding adjacent non‐tumour tissues (*P* < .05, Figure 1A). Likewise, the expression of miR‐1914 was obviously lower in the HCC cell lines than in the physiological liver cell line L02 (*P* < .05, Figure 1B). We selected MHCC‐97L cells (relatively low expression of miR‐1914) for miR‐1914 overexpression and Hep3B cells (relatively high expression of miR‐1914) for miR‐1914 knockdown in the following experiments. Furthermore, miR‐1914 expression was reduced in HCC tissues with a high histological grade (Edmondson‐Steiner III+IV, *P* < .05, Figure 1C) and large tumour size (≥5 cm, *P* < .05, Figure 1D).

### 3.2 miR‐1914 inhibits cell proliferation, cell cycle and colony formation and promotes apoptosis in HCC cells

**Figure 2:**
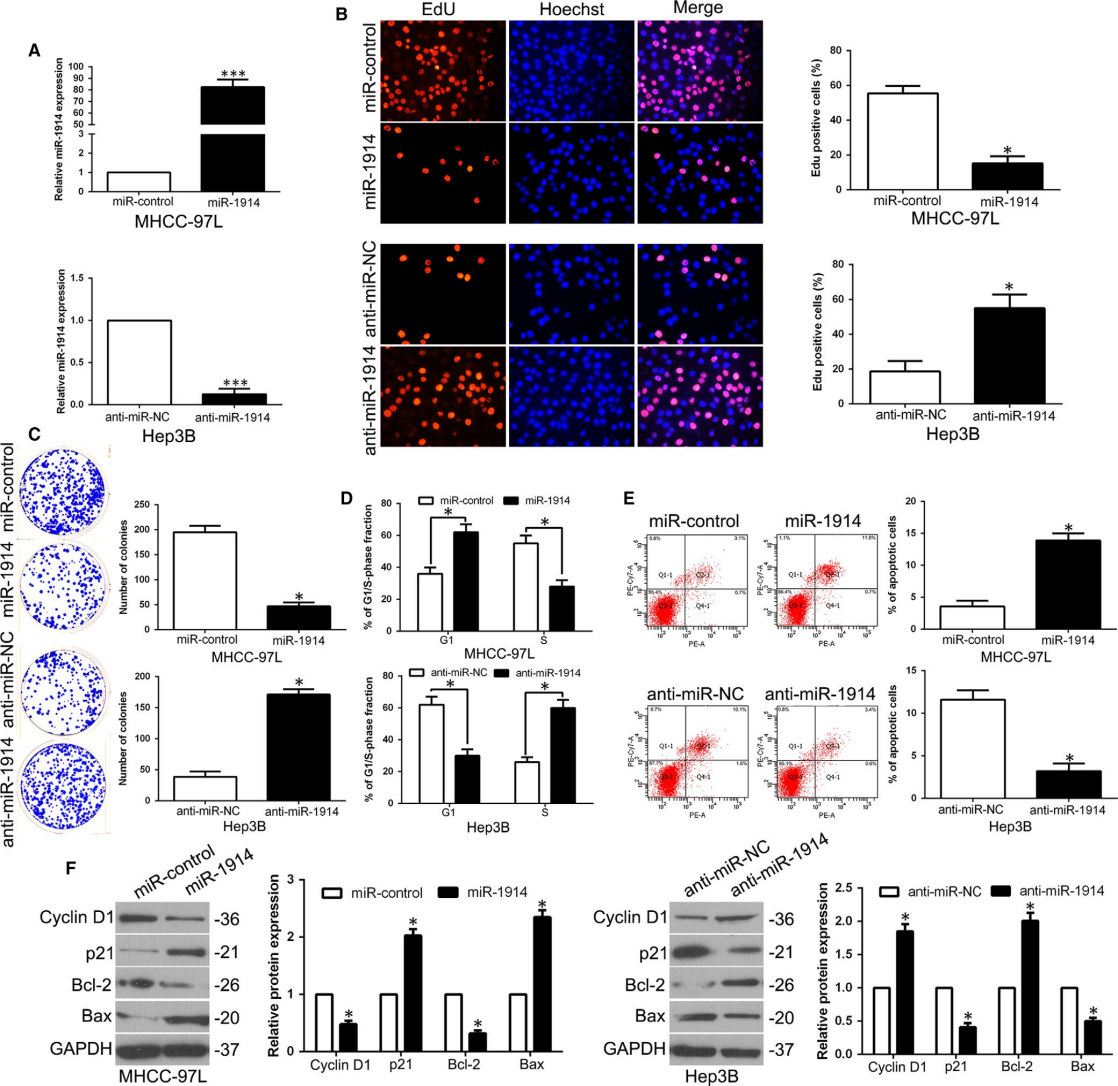
miR‐1914 inhibits cell proliferation, colony formation and cell cycle progression and promotes apoptosis in Hepatocellular carcinoma (HCC) cells in vitro. (A) We transfected with overexpression or depletion vectors of miR‐1914 into MHCC‐97L and Hep3B cells, and these cells underwent qRT‐PCR analysis for detecting miR‐1914 level. Up‐regulation of miR‐1914 inhibited proliferation (B), the ability of colony formation (C) and cell cycle progression (D) and promoted apoptosis (E) in MHCC‐97L cells. Reduced expression of miR‐1914 promoted cell proliferation (B), the capacity of colony formation (C) and cell cycle progression (D) and inhibited apoptosis (E) in Hep3B cells. (F) After up‐regulation or down‐regulation of miR‐1914, the expression of cell cycle–related proteins p21 and cyclin D1, and apoptosis regulators Bax and Bcl‐2 was determined by WB. n = six independent experiments. *P < .05, ***P < .001

To determine the efficacy of miR‐1914 in HCC, a series of functional experiments were conducted using a lentivirus system to stably overexpress miR‐1914 in MHCC‐97L cells and knock down miR‐1914 in Hep3B cells (Figure 2A, *P* < .05).

The results of EdU assays revealed that the overexpression of miR‐1914 remarkably suppressed the proliferation of MHCC‐97L cells (*P* < .05, Figure 2B). Moreover, the ectopic expression of miR‐1914 significantly inhibited the number of cell colonies, as confirmed by the colony formation assay (*P* < .05, Figure 2C). Moreover, as measured by flow cytometric analysis, the up‐regulation of miR‐1914 increased the numbers of G1 phase cells (*P* < .05, Figure 2D) and apoptotic cells (*P* < .05, Figure 2E). Additionally, the WB results confirmed that miR‐1914 overexpression obviously regulated the expression of cyclin D1, p21, Bcl‐2 and Bax (*P* < .05, Figure 2F). Nevertheless, miR‐1914 depletion promoted cell proliferation, colony formation and G1 phase arrest and inhibited apoptosis (*P* < .05, Figure 2B‐E). Cell cycle‐ and apoptosis‐related proteins showed corresponding effects (*P* < .05, Figure 2F).

**Figure 3:**
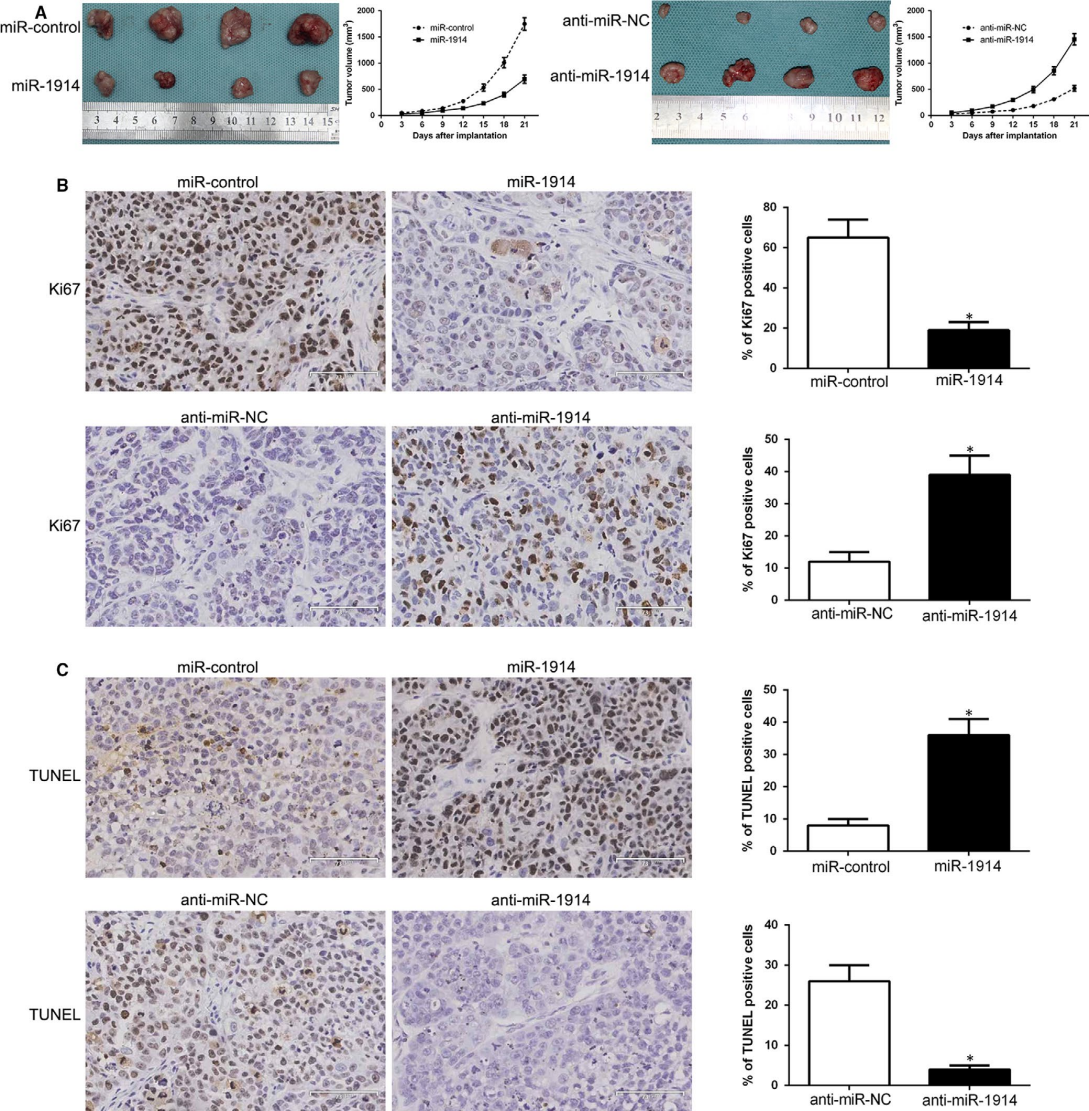
In vivo studies substantiate that miR‐1914 inhibits the growth of subcutaneously implanted tumours and promotes apoptosis. Representative images and tumour growth curve of subcutaneously implanted from both MHCC‐97L‐miR‐1914 and Hep3B‐anti‐miR‐1914 cells are displayed in (A). Tissue sections of tumours were subjected to Ki‐67 (B), TUNEL (C) assay and corresponding quantitative analysis by immunohistochemistry. * *P* < .05

To further determine the functional effects in vivo, we utilized the subcutaneous tumour growth model. Tumour growth curves showed significant inhibition of growth with miR‐1914 overexpression, whereas miR‐1914 depletion promoted tumour cell growth in vivo (*P* < .05, Figure 3A). We then performed Ki67 and TUNEL IHC staining in subcutaneous tumours to assess proliferation and apoptosis rates. Interestingly, miR‐1914 overexpression reduced the number of Ki67‐positive cells and increased the number of cells undergoing apoptosis according to TUNEL staining (*P* < .05, Figure 3B,C). Nevertheless, silencing miR‐1914 promoted growth in vivo and inhibited apoptosis (*P* < .05, Figure 3B,C).

### 3.3 GPR39 is recognized as a direct target gene of miR‐1914

**Figure 4:**
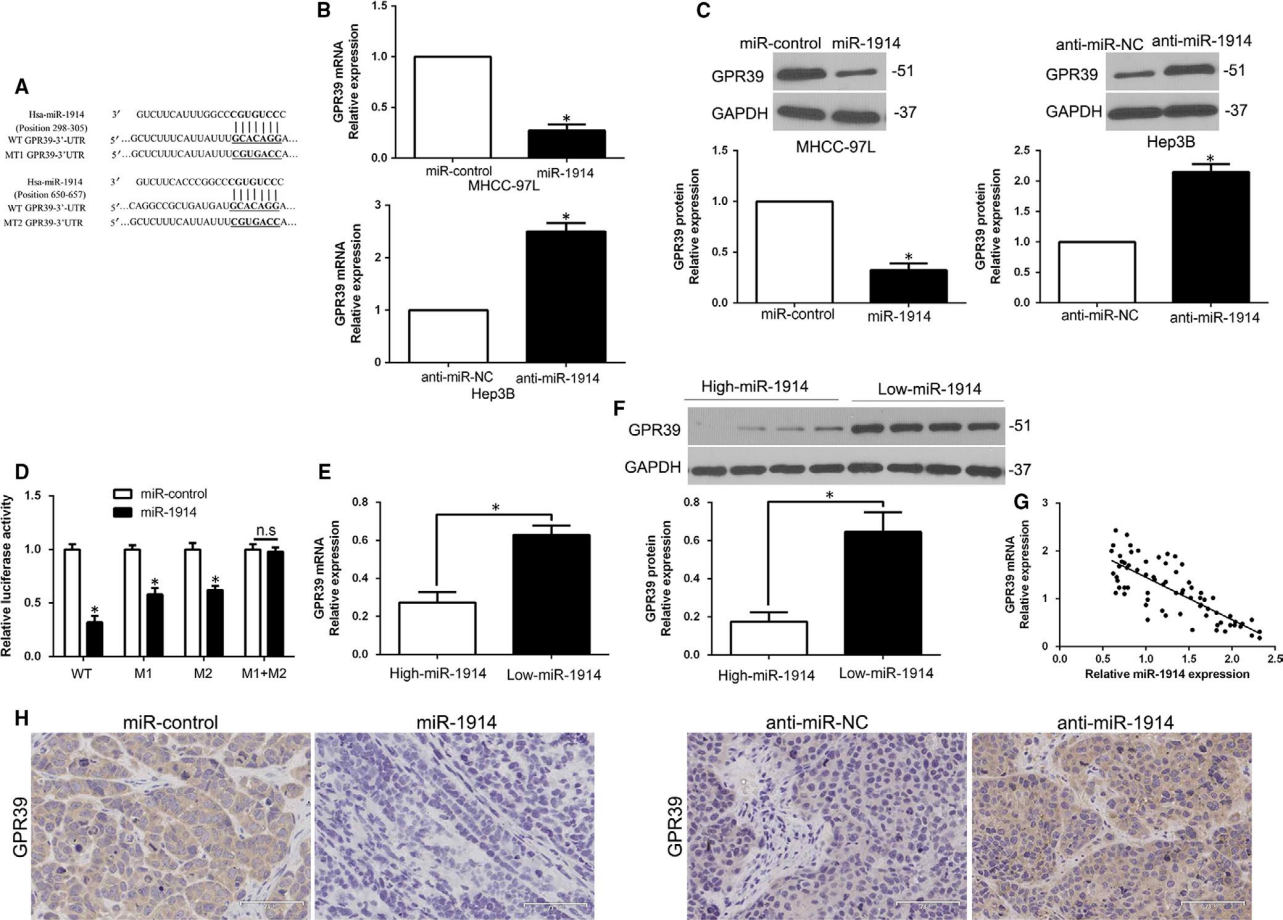
GPR39 is identified as a direct target with miR‐1914 in Hepatocellular carcinoma (HCC) cells. (A) The 3′‐UTR of GPR39 possessed the two binding sequences of miR‐1914, and the mutant binding sequences were synthesized and transfected into cells for the further experiments. (B) qRT‐PCR analysis to examine the mRNA level of GPR39 by MHCC‐97L transfected with the precursor miR‐1914 and Hep3B cells transfected with the miR‐1914 inhibitor (anti–miR‐1914). (C) miR‐1914 overexpression repressed the expression level of GPR39 in MHCC‐97L cells, and miR‐1914 knockdown enhanced the expression of GPR39 in Hep3B cells. (D) miR‐1914 overexpression remarkably restrained the luciferase activity in cells with wild‐type (wt) rather than mutant (mt) 3′‐UTR of GPR39. (E, F) GPR39 expression in miR‐1914–overexpressing tumours was obviously lower in comparison with that in tumours with depletion of miR‐1914. (G) A negative association between the expression of miR‐1914 and GPR39 level was found in HCC specimens. (H) IHC staining of GPR39 in subcutaneous tumour nodules with miR‐1914 overexpression or miR‐1914 depletion. n.s. represents no significance, *P < .05

Target algorithms (TargetScan, miRDB and StarBase v2.0) were utilized to uncover potential targets of miR‐1914. GPR39 was the predicted target (Figure 4A). To verify this, we examined the mRNA and protein levels of GPR39 by qRT‐PCR and WB assays and demonstrated that miR‐1914 significantly inversely regulated GPR39 expression (*P* < .05, Figure 4B,C). Similarly, a dual‐luciferase reporter assay showed that enhanced miR‐1914 expression effectively suppressed the relative luciferase activity of wild‐type (wt) GPR39 but not that of mutant (mt) GPR39 (*P* < .05, Figure 4D). Moreover, in clinical samples, we discovered that the expression of GPR39 was dramatically lower in high miR‐1914 expression tumours than in low miR‐1914 expression tumours (*P* < .05, Figure 4E,F). Additionally, we determined a dramatically negative association between miR‐1914 and GPR39 mRNA by Spearman's analysis in HCC samples (*P* < .05, Figure 4G). Furthermore, the expression of GPR39 in xenograft tissues was analysed, and the data indicated that GPR39 expression was higher in the miR‐1914 knockdown group than in the miR‐1914 overexpression group (*P* < .05, Figure 4H).

### 3.4 Characterization of GPR39 status in HCC

**Figure 5:**
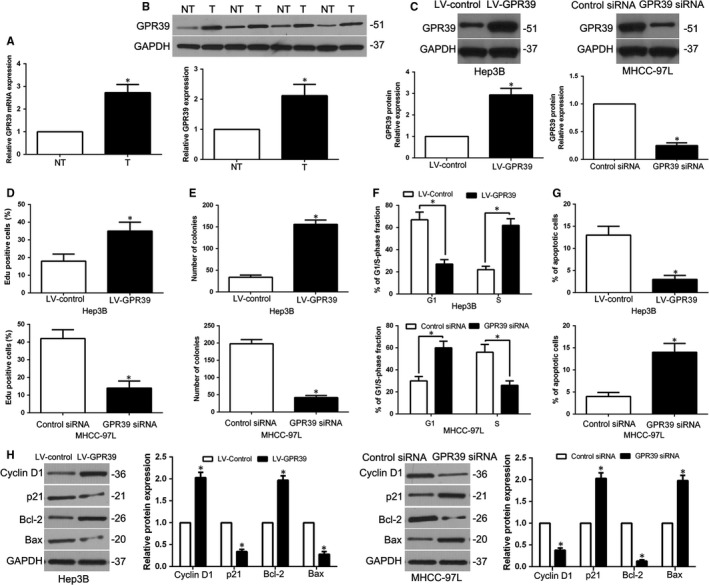
GPR39 was elevated in Hepatocellular carcinoma (HCC) and regulated the cell growth in HCC. (A, B) The GPR39 mRNA and protein level was up‐regulated in HCC tissues. (C) MHCC‐97L transfected with GPR39 siRNA and Hep3B cells transfected with LV‐GPR39 were examined in WB for GPR39 expression. GPR39‐overexpressing cells increased cell proliferation (D), colony formation (E) and cell cycle progression (F) and reduced apoptosis (G) in Hep3B cells, and depletion of GPR39 impeded proliferation (D), colony formation (E) and cell cycle progression (F) and enhanced apoptosis (G) in MHCC‐97L cells. (H) After up‐regulation or down‐regulation of GPR39, the expression of cell cycle–related proteins cyclin D1 and p21, and apoptosis regulator Bax and Bcl‐2 was determined by WB. n = six independent experiments. *P < .05

To investigate the level of expression and the potential role of GPR39 in HCC tissues, we conducted qRT‐PCR and WB assays, and the results showed that GPR39 levels were much lower in adjacent non‐tumour tissues than in HCC tissues (*P* < .05, Figure 5A,B). Moreover, we established stable GPR39 overexpression or knockdown cells (*P* < .05, Figure 5C) by lentiviral transduction. EdU assays showed that GPR39 overexpression significantly promoted the proliferation of Hep3B cells (*P* < .05, Figure 5D). The ability of colony formation confirmed that GPR39 up‐regulation significantly increased the number of cell colonies (*P* < .05, Figure 5E). Moreover, flow cytometric analysis showed that the up‐regulation of GPR39 resulted in cell cycle progression (*P* < .05, Figure 5F) and decreased apoptosis (*P* < .05, Figure 5G). Additionally, WB confirmed that GPR39 overexpression obviously regulated the cell cycle–related proteins cyclin D1 and p21 and the apoptosis‐related proteins Bcl‐2 and Bax (*P* < .05, Figure 5H). In contrast, GPR39 silencing impeded cell proliferation and colony formation capacity, and induced G1 phase accumulation, as well as enhanced apoptosis (*P* < .05, Figure 5D‐G). Cell cycle‐ and apoptosis‐related proteins showed corresponding effects (*P* < .05, Figure 5H). Therefore, we illustrated that GPR39 alteration could mimic the miR‐1914–induced effects in HCC cells.

### 3.5 Restoration of GPR39 expression partially reversed the miR‐1914–induced biological effects in HCC cells

**Figure 6:**
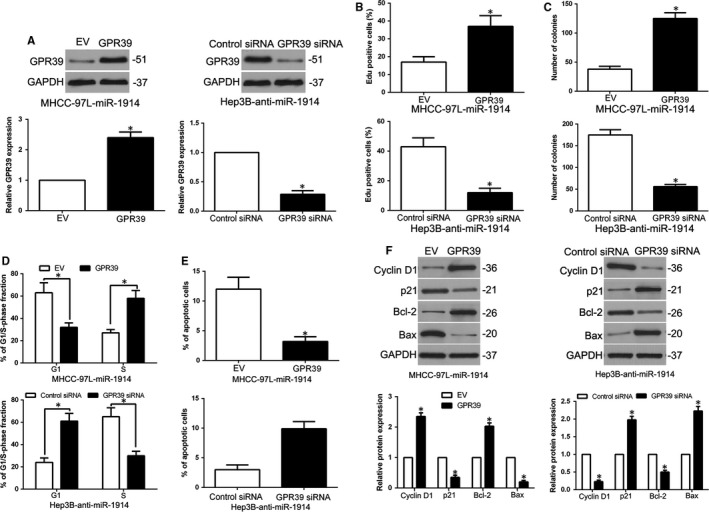
Mediation of GPR39 partially reverses the miR‐1914–mediated cellular functions in Hepatocellular carcinoma (HCC). (A) We transfected overexpression plasmid of GPR39 into miR‐1914–overexpressing MHCC‐97L cells, and GPR39 siRNA into miR‐1914–suppressive Hep3B cells. The expression level of GPR39 was assessed by WB. GPR39 reversion remarkably abolished the efficacy of miR‐1914 overexpression on cell proliferation (B), the ability of colony formation (C), cell cycle progression (D) and apoptosis (E) of MHCC‐97L cells. GPR39 depletion reversed the promoting efficacy of Hep3B cells with miR‐1914 silence (B‐E). (F) Alteration of GPR39 expression erased the efficacy of miR‐1914 on apoptosis and cell cycle correlated regulators^,^ expression. n = 6 independent experiments. *P < .05

For further investigate whether GPR39 was a functional target of miR‐1914, we overexpressed GPR39 in miR‐1914–overexpressing MHCC‐97L cells (*P* < .05, Figure 6A); GPR39 restoration abolished the effects of miR‐1914, leading to remarkable increases in proliferation, colony formation, G1 phase arrest and apoptosis inhibition in miR‐1914–overexpressing MHCC‐97L cells (*P* < .05, Figure 6B‐F). In addition, GPR39 knockdown in miR‐1914–silenced Hep3B cells partly inhibited the effects of anti–miR‐1914 on tumour cell proliferation, colony formation, cell cycle progression and apoptosis (*P* < .05, Figure 6A‐F). Thus, the data showed that GPR39 was a functional target of miR‐1914 in HCC.

### 3.6 miR‐1914 and GPR39 expression in HCC indicates the prognosis

**Figure 7:**
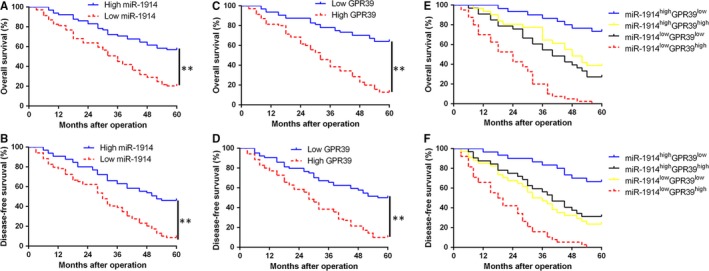
The miR‐1914 and GPR39 show prognostic value for Hepatocellular carcinoma (HCC) patients. (A, C) and (B, D) OS and DFS were compared between HCC patients with miR‐1914 high expression and low‐expressing cases, or with GPR39 high expression and low‐expressing cases, respectively. (E) and (F) OS and DFS were compared between four subgroups of HCC cases (subgroup I: high miR‐1914/low GPR39; subgroup II: high miR‐1914/low GPR39; subgroup III: low miR‐1914/high GPR39; and subgroup IV: low miR‐1914/high GPR39). For each cohort, subgroups were divided based on the median value of the relative expression of miR‐1914 and GPR39 in HCC tissues. **P < .01

To confirm the clinical use of miR‐1914 and GPR39 in HCC, we investigate their correlation with clinical features. As shown in Table 1, miR‐1914 down‐regulation was dramatically related to large tumour size (≥5 cm; *P* = .011), high histological grade (Edmondson‐Steiner grade III+IV; *P* = .002) and advanced tumour stage (TNM stage III+IV; *P* = .023), whereas GPR39 overexpression was correlated with large tumour size (*P* = .004) and high histological grade (*P* = .028). Moreover, a Kaplan‐Meier survival analysis showed that HCC patients with miR‐1914 down‐regulation had a worse OS (overall survival) and DFS (disease‐free survival), whereas patients with high GPR39 expression possessed a poorer DFS and OS (*P* < .05, Figure 7A‐D). Through a combination analysis, we demonstrated that concurrently decreased miR‐1914 and increased GPR39 levels were associated with the shortest OS and DFS (*P* < .05, Figure 7E,F).

**Table 1:** Correlation between the clinicopathologic characteristics and miR‐1914 and GPR39 expression in HCC (n = 134)

Clinical parameters	Cases	Expression level		*P* value	Expression level		*P* value
		miR‐1914^high^ (n = 65)	miR‐1914^low^ (n = 69)		GPR39^high^ (n = 70)	GPR39^low^ (n = 64)	
Age (y)							
<65 y	71	33	38	.618	35	36	.469
≥65 y	63	32	31		35	28	
Gender							
Male	108	52	56	.865	56	52	.855
Female	26	13	13		14	12	
Tumour size (cm)							
＜5 cm	105	57	48	.011*	48	57	.004*
≥5 cm	29	8	21		22	7	
Tumour number							
Solitary	116	57	59	.711	60	56	.762
Multiple	18	8	10		10	8	
Edmondson							
Ⅰ+Ⅱ	97	55	42	.002*	45	52	.028*
Ⅲ+Ⅳ	37	10	27		25	12	
TNM stage							
Ⅰ+Ⅱ	109	58	51	.023*	56	53	.676
Ⅲ+Ⅳ	25	7	18		14	11	
Capsular							
Present	93	46	47	.739	46	47	.333
Absent	41	19	22		24	17	
Venous invasion							
Present	17	8	9	.898	10	7	.561
Absent	117	57	60		60	57	
AFP							
＜400 ng/mL	36	16	20	.568	19	17	.940
≥400 ng/mL	98	49	49		51	47	
HBsAg							
Positive	120	59	61	.655	63	57	.859
Negative	14	6	8		7	7	

Abbreviations: AFP, alpha‐fetoprotein; HCC, hepatocellular carcinoma; TNM, tumour‐node‐metastasis.

* *P* < .05.

### 3.7 AKT phosphorylation plays a crucial role in PI3K/AKT/mTOR signalling mediated the biological effects of miR‐1914 in HCC

**Figure 8:**
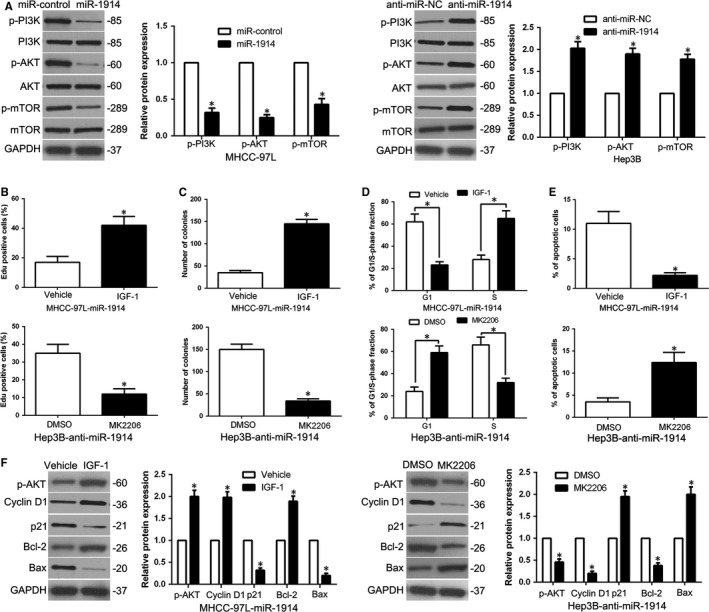
AKT phosphorylation is crucial to exert the physiological efficacy of miR‐1914 in Hepatocellular carcinoma (HCC). (A) After transfected with corresponding miRNA vectors into MHCC‐97L and Hep3B cells, respectively, these cells underwent immunoblotting analysis to detect the expression of PI3K/AKT/mTOR. MK2206 (an inhibitor of AKT), or IGF‐1 (AKT phosphorylation activator), remarkably abrogated the cell proliferation (B), the capacity of colony formation (C), the progression of cell cycle (D) and the rate of apoptosis (E) in HCC cells which were transfected with miR‐1914 vectors. (F) The result of WB verified that modulation of AKT phosphorylation obviously erased the efficacy of miR‐1914 alteration on the expression of apoptosis‐associated regulators in HCC cells. n = 6 independent experiments. *P < .05

To explore the molecular mechanisms that mediate the efficacy of miR‐1914, we examined PI3K/AKT/mTOR signalling using a WB assay. As shown in Figure 8A, miR‐1914 overexpression dramatically decreased p‐AKT and mTOR levels, whereas miR‐1914 silencing increased p‐AKT and mTOR levels in HCC cells. As previously reported,^20^, ^21^ IGF‐1, an AKT activator, plays a vital role in the activation of the PI3K/AKT pathway. To determine whether AKT phosphorylation mediated the effects of miR‐1914, an inhibitor (MK2206) and activator (IGF‐1) of AKT were applied to modulate the status of AKT phosphorylation. The miR‐1914–overexpressing MHCC‐97L cells were treated with IGF‐1, and IGF‐1 intervention partially abolished the miR‐1914–induced effects on cell proliferation, colony formation, cell cycle and apoptosis (*P* < .05, Figure 8B‐F). Conversely, the suppression of AKT phosphorylation by MK2206 abolished the function of miR‐1914 depletion in Hep3B cells (*P* < .05, Figure 8B‐F).

### 3.8 miR‐1914 is negatively modulated by lncRNA DUXAP10 in HCC cells

**Figure 9:**
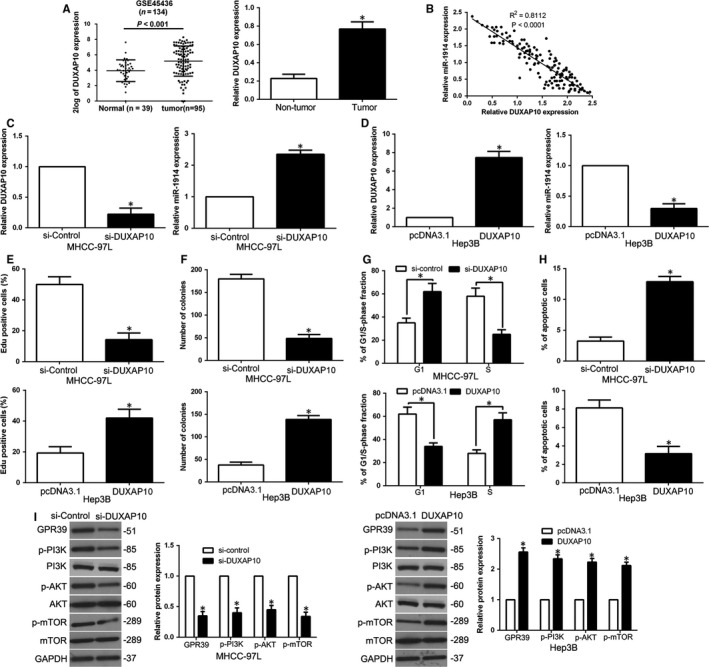
miR‐1914 is negatively modulated by lncRNA DUXAP10 in Hepatocellular carcinoma (HCC) cells. (A) lncRNA DUXAP10 expression differences existed in HCC samples and neighbouring non‐tumour specimens. (B) The level of miR‐1914 was negatively correlated to DUXAP10 in HCC tissues. (C) MHCC‐97L cells carried with DUXAP10 siRNA underwent the qRT‐PCR analysis. DUXAP10 depletion markedly elevated miR‐1914 level in MHCC‐97L cells. (D) Overexpression of DUXAP10 greatly restrained the level of miR‐1914 in Hep3B cells. Down‐regulation of DUXAP10 suppressed proliferation (E), colony formation (F) and the progression of cell cycle (G) and facilitated apoptosis (H) in MHCC‐97L cells, whereas overexpression of DUXAP10 facilitated cell proliferation (E), colony formation (F) and the progression of cell cycle (G) and restrained apoptosis (H) in Hep3B cells. (I) Analysis of downstream GPR39/PI3K/AKT protein in MHCC‐97L with or without depletion of DUXAP10 was confirmed by WB. n = 6 independent experiments. *P < .05

To better elucidate the underlying mechanism by which miR‐1914 was depressed in HCC, we predicted the potential targets using Starbase v2.0 and discovered that lncRNA DUXAP10 is a molecular sponge that may regulate miR‐1914. First, we used the GEO database to show that DUXAP10 was obviously higher in HCC samples than in normal liver tissues, and we confirmed that DUXAP10 was higher in HCC tissues than in neighbouring non‐tumour tissues (Figure 9A, *P* < .05). Spearman correlation analysis revealed a negative correlation between DUXAP10 expression and miR‐1914 levels in HCC specimens (*R* ^2^ = 0.8112, *P* < .05, Figure 9B). Additionally, DUXAP10 expression was knocked down with an siRNA sequence, and DUXAP10 depletion increased the level of miR‐1914 in MHCC‐97L cells (*P* < .05, Figure 9C), whereas DUXAP10 overexpression suppressed the expression of miR‐1914 in Hep3B cells (*P* < .05, Figure 9D). Moreover, we explored whether DUXAP10 modulated biological effects and GPR39‐mediated PI3K/AKT signalling in HCC cells. Amazingly, DUXAP10 knockdown inhibited proliferation, colony formation ability and G1 phase arrest and increased apoptosis (*P* < .05, Figure 9E‐H). Moreover, DUXAP10 knockdown inhibited GPR39 expression and PI3K/AKT signalling in MHCC‐97L cells (*P* < .05, Figure 9I). Conversely, DUXAP10 overexpression promoted cell proliferation, colony formation, cell cycle progression and PI3K/AKT signalling and inhibited apoptosis in Hep3B cells (Figure 9E‐I).

## 4 DISCUSSION

To date, accumulative evidence has validated that abnormal miRNA expression is pivotal in HCC carcinogenesis and progression.^22^ Numerous studies have established the potential usefulness of miRNAs as therapeutic molecules in cancers.^23^, ^24^ Herein, our study confirmed that the level of miR‐1914 was remarkably decreased in HCC specimens and cells. In addition, miR‐1914 was down‐regulated in tumours with a large size and high histological stage. These findings verify that miR‐1914 serves as a tumour suppressor in HCC development.

Previous studies confirmed that miRNAs serve as prognostic biomarkers and valid pharmaceutical targets for HCC.^25^, ^26^ In the present study, we corroborate that miR‐1914 impedes cell proliferation, induces cell cycle arrest and exacerbates apoptosis through a series of gain‐ and loss‐of‐function assays. Furthermore, GPR39 is a direct target gene of miR‐1914. miR‐1914 inhibits GPR39 expression in HCC cells. miR‐1914 blocks the luciferase activity of the wt 3'‐UTR of GPR39 and is negatively correlated with GPR39 expression in HCC tissues. We also confirmed that GPR39 expression was enhanced in HCC tissues. GPR39 stimulated HCC cell growth. Moreover, altering GPR39 levels could partially rescue the physiological roles of miR‐1914 in HCC cells. GPR39, a G protein‐coupled receptor activated by zinc, controls proliferation and differentiation in different cells.^27^, ^28^, ^29^ GPR39 is also dysregulated in cancers.^30^, ^31^, ^32^ Here, we showed that miR‐1914 inhibited PI3K/AKT/mTOR signalling. Modulation of AKT phosphorylation could reverse the effects of miR‐1914 on HCC cells. Therefore, miR‐1914 exerts its functions by targeting GPR39 to inhibit PI3K/AKT/mTOR signalling. In addition, we found that the level of lncRNA DUXAP10 was higher in HCC tissues than in adjacent non‐tumour samples and was conversely related to the miR‐1914 expression level in HCC tissues. Subsequently, DUXAP10 negatively modulated miR‐1914 and promoted GPR39‐mediated PI3K/AKT signalling in HCC cells. According to clinical data, we confirmed that as valuable biomarkers, miR‐1914 and GPR39 could provide new insight into prognostic prediction. We illustrated that low miR‐1914 levels and high GPR39 levels were markedly correlated with poor clinical characteristics in HCC patients. Additionally, we demonstrated that the combination of low miR‐1914 expression and high GPR39 expression remarkably correlated with the adverse prognosis of patients suffering from HCC. These data indicated that miR‐1914 and GPR39 could be used as promising prognostic predictors of HCC.

Overall, we reported that miR‐1914 is decreased in HCC samples and cell lines, although miR‐1914 inhibits cell growth by directly targeting the GPR39‐mediated PI3K/AKT/mTOR signalling pathway. Notably, miR‐1914 reduction and GPR39 overexpression were associated with malignant clinicopathological features, and their combination could be used as a novel prognostic predictor for HCC patients. Furthermore, overexpression of lncRNA DUXAP10 regulates miR‐1914 expression, promotes cell proliferation and reduces the apoptosis rate via regulating GPR39/PI3K/AKT signalling in HCC cells. Overall, down‐regulation of miR‐1914 may be critical in regulating tumour growth and thus work as a valuable prognostic marker and novel therapeutic target for HCC.

## CONFLICT OF INTEREST

No conflicts of interest exist.

## AUTHOR CONTRIBUTIONS

WY and ZL conceived and designed the experiments; LS, LW, TC, BY and YW performed the experiments; QL analysed the data; and LS, WY and ZL wrote the paper. All authors read and approved the final manuscript.

## Data Availability

The data that support the findings of this study are available from the corresponding author upon reasonable request.
